# Violation of the 12/23 rule of genomic V(D)J recombination is common in lymphocytes

**DOI:** 10.1101/gr.179770.114

**Published:** 2015-02

**Authors:** Nicholas J. Parkinson, Matthew Roddis, Ben Ferneyhough, Gang Zhang, Adam J. Marsden, Siarhei Maslau, Yasmin Sanchez-Pearson, Thomas Barthlott, Ian R. Humphreys, Kristin Ladell, David A. Price, Chris P. Ponting, Georg Hollander, Michael D. Fischer

**Affiliations:** 1 Systems Biology Laboratory UK, Abingdon, Oxfordshire OX14 4SA, United Kingdom;; 2 MRC Functional Genomics Unit, Department of Physiology, Anatomy and Genetics, University of Oxford, Oxford OX1 3PT, United Kingdom;; 3 Paediatric Immunology, Department of Biomedicine, University of Basel and The Basel University Children’s Hospital, 4058 Basel, Switzerland;; 4 Institute of Infection and Immunity, Cardiff University School of Medicine, Heath Park, Cardiff CF14 4XN, United Kingdom;; 5 Vaccine Research Center, National Institute of Allergy and Infectious Diseases, National Institutes of Health, Bethesda, Maryland 20892, USA;; 6 Developmental Immunology, Weatherall Institute of Molecular Medicine and Department of Paediatrics, University of Oxford, Oxford OX3 9DS, United Kingdom;; 7 Department of Oncology, Division of Cellular and Molecular Medicine, St. George’s, University of London, London SW17 0QT, United Kingdom

## Abstract

V(D)J genomic recombination joins single gene segments to encode an extensive repertoire of antigen receptor specificities in T and B lymphocytes. This process initiates with double-stranded breaks adjacent to conserved recombination signal sequences that contain either 12- or 23-nucleotide spacer regions. Only recombination between signal sequences with unequal spacers results in productive coding genes, a phenomenon known as the “12/23 rule.” Here we present two novel genomic tools that allow the capture and analysis of immune locus rearrangements from whole thymic and splenic tissues using second-generation sequencing. Further, we provide strong evidence that the 12/23 rule of genomic recombination is frequently violated under physiological conditions, resulting in unanticipated hybrid recombinations in ∼10% of *Tcra* excision circles. Hence, we demonstrate that strict adherence to the 12/23 rule is intrinsic neither to recombination signal sequences nor to the catalytic process of recombination and propose that nonclassical excision circles are liberated during the formation of antigen receptor diversity.

The adaptive immune system recognizes a seemingly unlimited array of antigens via a highly diverse repertoire of B and T cell antigen receptors (Sakano et al. 1979; Tonegawa 1983; Davis and Bjorkman 1988). These heterodimeric molecules contain a variable antigen-binding domain encoded through recombination of variable (V), diversity (D, only present in some loci), and joining (J) gene segments. Each gene segment is flanked by a canonical recombination signal sequence (RSS) composed of conserved heptamer and nonamer motifs separated by a less well-conserved spacer of either 12 or 23 base pair (bp) in length (for review, see Schatz and Ji 2011). V(D)J recombination is initiated by the lymphocyte-specific recombination activating gene (RAG) recombinase, a complex mainly composed of RAG1 and RAG2 proteins. The RAG recombinase introduces double-stranded breaks at RSS sites, resulting in the generation of covalently sealed hairpins at gene segment ends. Before re-ligation, these DNA ends may be processed further to produce local deletions or nontemplated nucleotide (N base) additions. Multiple enzymes, including components of the nonhomologous end joining (NHEJ) complex, are involved in the processing and repair of the final genomic coding junction (Fig. 1A). In parallel, a signal junction is generated by ligating the remaining DNA ends with few sequence modifications to form an excision circle (EC). Recombination between 12-bp and 23-bp RSSs, the 12/23 rule, ensures that productive coding rearrangements are formed from V, D, and J gene segments (Fig. 1A). Although junctions have been reported that do not keep to the 12/23 rule (Mansikka and Toivanen 1991; Shimizu et al. 1991), these are either largely confined to nonphysiological recombination events in the absence of regular RAG (Talukder et al. 2004) or NHEJ (Bogue et al. 1997; Han et al. 1997) complex expression, or they are triggered by cryptic RSS sequences (Davila et al. 2007). Non-12/23 junctions under physiological conditions are thought to be rare, the most common being in VDDJ rearrangements of the *Igh* locus that occur once per ∼800 cells (Briney et al. 2012). In addition to violating the 12/23 rule, other noncanonical rearrangements form hybrid signal-to-coding junctions. These are typically generated in artificial systems (Lewis et al. 1988; Morzycka-Wroblewska et al. 1988; Alexandre et al. 1991; Bogue et al. 1997; Han et al. 1997; Melek et al. 1998; Bredemeyer et al. 2006; Briney et al. 2012) but may also be detected at low frequency in vivo under physiological conditions (Alexandre et al. 1991; Carroll et al. 1993; Sollbach and Wu 1995; VanDyk et al. 1996).

**Figure 1.**
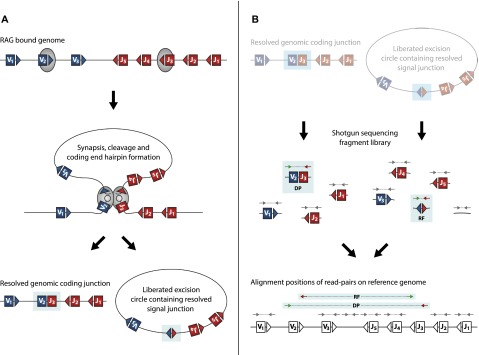
Schematic representation of RAG-mediated V(D)J recombination showing the relative mapping positions of DP and RF read-pairs from recombined genomic and excised circular DNA. (*A*) Hypothetical genomic locus containing variable (blue box) and joining (red box) elements flanked by recombination signal sequence (RSS) motifs (blue and red triangles) are bound and brought into close association by the recombination activating gene (RAG) complex (gray). RAG-mediated double-stranded cleavage at RSS sites precedes genomic deletion (coding) end processing by the nonhomologous end joining (NHEJ) complex. Coding ends are covalently hair-pinned (gray circle section) and reopened prior to ligation. Secondarily, the excision circle (EC; signal) junction is ligated from unprocessed DNA ends with little modification. (*B*) Sequencing library fragments from recombined EC and genomic (g) DNA are shown. Read-pair sequences from fragments are mapped back to the reference genome, allowing junction-spanning reads to be identified. Gray arrows represent “standard” read-pairs generated from EC DNA or gDNA and map to the reference genome in standard orientation separated by a mapping distance equal to the initial fragment length. Deletion read-pairs (DPs) spanning a coding junction map to the reference genome with standard relative orientation—the forward read (green) mapping 5′ of and facing the reverse read (red)—but are separated by mapping distances greater than the initial fragment length. Reverse-forward read-pairs (RFs) spanning the signal junction map to the reference genome in an inverted relative read orientation so that the reverse read (red) maps 5′ of and faces away from the forward read (green). RF read-pairs are separated by the full length of the circle from which they originate rather than the initial library fragment length.

Most studies of V(D)J recombination have been limited to PCR amplification and Sanger sequencing of predefined coding, signal, or transgenic junctions. Second-generation sequencing technologies, which allow an unbiased capture of rearrangements, have only been used to study V(D)J recombination in T cell receptor (TCR)–derived mRNA sequences (Genolet et al. 2012). This approach is limited to analyses of coding junctions in productive, successfully expressed mRNAs and excludes quantitative insights into genomic recombination frequencies.

We have developed two complementary techniques, excision circle (EC)-seq and immune region (IR)-seq, for direct second-generation sequencing analyses of V(D)J rearrangements in EC and chromosomal DNA. Using these techniques, we report that an abundance of unanticipated J-J and, to a lesser extent, V-V excision circles is produced under physiological conditions at multiple antigen receptor chain loci in both mice and humans, thus both violating the 12/23 rule of recombination and resulting in coding-to-signal hybrid junctions. Our data suggest that adherence to the 12/23 rule is not intrinsic to RSS motifs or the RAG recombinase but are conferred by additional factors and that these high-frequency nonclassical rearrangements are liberated during production of a diverse immune repertoire.

## Results

### EC-seq captures classical Vα-Jα junctions

**Figure 2.**
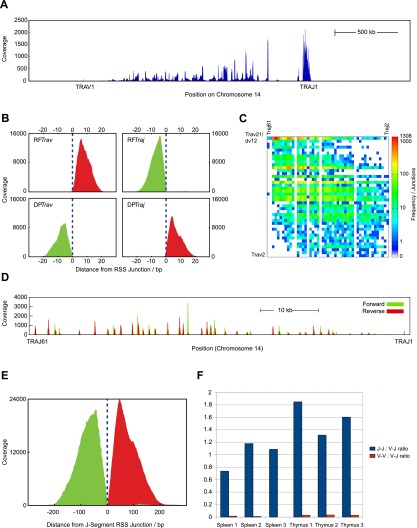
EC-seq enriches *Tcra* locus-associated circularized excision material. (*A*) Gross coverage of perfectly aligned read-pairs (AA) across the mouse *Tcra* locus on Chromosome 14 in whole thymus-captured EC-seq material. (*B*) Meta-analysis of Vα-Jα RF read-pairs (*top*) or DP read-pairs (*bottom*) in EC-seq libraries showing close association of reads with known Vα element subregion RSSs (*left*) or Jα element subregion RSSs (*right*). The data set comprises thymic and splenic EC-seq replicates. Overlay of forward reads is shown in green; overlay of reverse reads is shown in red. Broken vertical lines indicate the RSS cleavage site. (*C*) Heatmap analysis of 31,535 RSS-associated Vα-Jα RF junctions from EC-seq metadata displaying the diversity of recombined Vα-Jα ECs. Data are from three thymic and three splenic replicates. ECs are only shown for active elements with uniquely mappable sequences. (*D*) EC-seq pile-up of Jα-Jα RF read-pairs across the Jα segment subregion showing clustering of Jα element RSSs. Forward reads are shown in green; reverse reads are shown in red. (*E*) Meta-analysis of Jα-Jα subregion RFs relative to known Jα region RSS sites. Forward reads are shown in green; reverse reads are shown in red. (*F*) Ratio of Jα-Jα (blue bars) and Vα-Vα (red bars) to Vα-Jα RFs in three thymic and three splenic EC-seq libraries.

DNA extracts enriched for extra-chromosomal mouse excision circles were used to produce second-generation sequencing libraries and resultant data aligned to the mouse genome (see Methods). Replicate thymic or splenic EC samples were greatly enriched for standard read-pairs mapping across B and T cell antigen receptor loci, including the TCR αδ chain locus, *Tcra* (440 ± 80-fold for thymus samples and 50 ± 15-fold for spleen samples) (Supplemental Table 1). Reverse-forward read-pairs (RFs; as defined and illustrated in Fig. 1B) span the ligated signal junction of excision circles and can be readily identified (see Methods). RFs were substantially enriched across the *Tcra* locus (3643 ± 1614-fold for thymus and 249 ± 173-fold for spleen) (Supplemental Table 1). Most *Tcra* RFs (95.7 ± 0.7%) mapped within 300 bp of a recognized RSS site (Supplemental Table 2) in the expected sequence architecture for Vα-Jα ECs: R reads aligned 3′ of the Vα RSS element, and F reads aligned 5′ of the Jα RSS element (Figs. 1B, 2). In total, we recovered 59,632 ± 15,964 RSS-associated Vα-Jα ECs from each thymus and 2957 ± 1238 RSS-associated Vα-Jα ECs from each spleen (Supplemental Table 2). Heatmap analysis of RF read-pairs demonstrated that the EC data set contained a highly diverse repertoire of Vα-Jα recombinations displaying preferential usage of 3′ Vα- and 5′ Jα-gene segments across the *Tcra* locus (Fig. 2C).

Deletion read-pairs (DPs) span the coding ligation site created by a recombinational excision event (illustrated in Fig. 1B). EC-seq libraries of both thymic and splenic T cells contained abundant DP read-pairs. Most *Tcra* DPs (96 ± 3%) mapped within 300 bp of a known RSS site (Fig. 2; Supplemental Table 2) with the expected sequence architecture: F reads aligned 5′ of the Vα RSS element, and R reads aligned 3′ of the Jα RSS element (Fig. 2B). We recovered 19,913 ± 2888 RSS-associated Vα-Jα DPs from each thymus and 1202 ± 475 RSS-associated Vα-Jα DPs from each spleen (Supplemental Table 2). In contrast, neither RF nor DP read-pairs were enriched in EC DNA isolated from the thymus or spleen of *Rag1*-deficient mice (Supplemental Table 1). Hence, the captured excision events are the result of RAG-mediated V(D)J recombinations.

### EC-seq captures nonclassical Jα-Jα junctions

**Figure 3.**
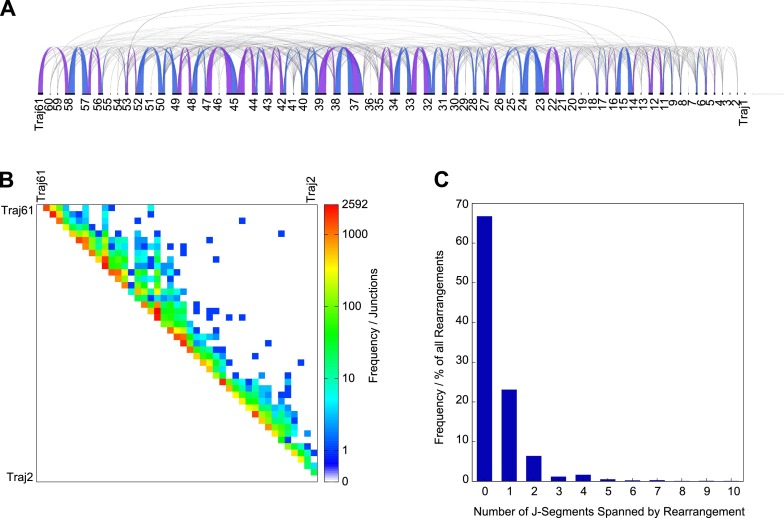
Jα-Jα ECs occur predominantly with their nearest neighbor. (*A*) Graphical representation of individual Jα-Jα ECs from a whole thymus EC-seq library showing connectivity and relative frequency of Jα-Jα ECs across the Jα element subregion. Ribbon width represents the frequency of recombinations between connected segments. Ribbon color is alternated to discriminate between independent ribbon connections at the same segment. (*B*) Heatmap meta-analysis of 33,332 Jα-Jα RFs showing the frequency of individual EC events. (*C*) Jα-Jα EC events from thymic and splenic EC-seq libraries showing the frequency of excisions spanning neighboring or multiple genomic Jα segments.

EC-seq data sets also contained abundant RFs for which both reads mapped adjacent to Jα gene segment RSSs (15,700 ± 5000 in thymus, 640 ± 310 in spleen) (Fig. 2D,E). This finding was reproduced in all three thymic and splenic EC-seq libraries and revealed a frequency of putative Jα-Jα ECs equivalent to that of Vα-Jα ECs (Fig. 2F). Sixty-seven percent of Jα-Jα events occurred between immediately neighboring Jα gene segments with a marked bias in their use (Fig. 3). Analyses to correlate predicted RSS recombination signal information content (RIC) strength (Cowell et al. 2002) with frequency of element use detected no obvious relationship (data not shown). To further verify our findings, PCR amplicons from thymus EC DNA were generated using oligonucleotide primers designed across seven high-frequency candidate Jα-Jα combinations. Sequenced amplicons aligned with high specificity to all candidate junctions tested. As expected, brain EC DNA and liver genomic DNA failed to yield products. In-depth analysis of individual Jα-Jα junctions showed a large degree of sequence diversity at ligation sites, indicating that products were amplified from multiple individual EC junctions (Supplemental Fig. 1). Like Vα-Jα ECs, the generation of Jα-Jα RF read-pairs was RAG dependent, as they were not detected in *Rag1*-deficient thymic or splenic EC-seq libraries. Hence, Jα-Jα RFs represent circularization junctions from ECs formed predominantly from neighboring Jα gene segments and are highly abundant in thymocytes and peripheral T cells under physiological conditions.

### IR-seq confirms Jα-Jα excisions

The EC DNA extraction method may favor the capture of small circular molecules as large circular DNA species are more prone to exonuclease digestion and exclusion during purification over silica columns. Jα-Jα ECs are predicted from the genomic sequence to be smaller (1–10 kb) than primary or secondary Vα-Jα ECs (10 kb–1 Mb) and thus are likely enriched by our extraction technique.

**Figure 4.**
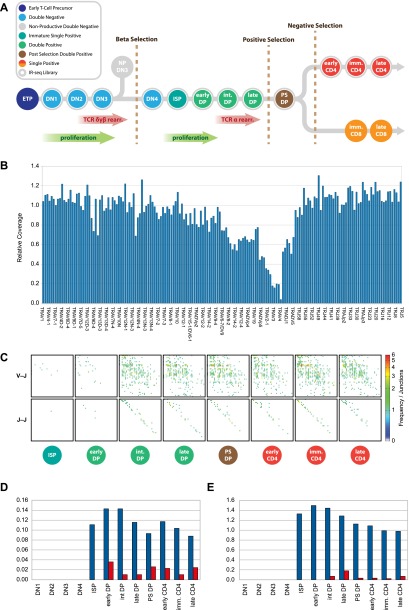
IR-seq analysis of T cell developmental stages confirms that Jα-Jα ECs originate during *alpha* chain rearrangement. (*A*) Schematic diagram of T cell development showing stage-specific, thymus-derived cell sorts used to generate 12 IR-seq libraries (see Methods). (*B*) Coverage in the intermediate CD4 single positive (ISP) IR-seq library across the *Tcra* locus. Coverage was computed between neighboring Vα, Dα, and Jα gene segment RSSs and normalized relative to coverage in the same regions in the prerecombinational DN1 IR-seq data set. Depleted coverage across the *Tcr delta* locus is predicted to result from genomic excision events and loss of *delta* ECs during preceding proliferative phases. Coverage across the Jα subregion remains at ∼1, indicating that Jα-Jα ECs are not overrepresented in IR-seq. (*C*) Heatmap analyses of sorted T cell precursors showing Vα-Jα EC events (*top*) and Jα-Jα EC events (*bottom*) for eight consecutive recombinationally active IR-seq libraries. (*D*) Jα-Jα–to–Vα-Jα (blue bars) and Vα-Vα–to–Vα-Jα (red bars) ratios of RFs in 12 IR-seq libraries, representing key stages of T cell development. (*E*) DP-to-RF ratios for Vα-Jα (blue bars) and Jα-Jα (red bars) in 12 IR-seq libraries representing key stages of T cell development.

To assess the relative abundance of Jα-Jα and Vα-Jα recombinations during T cell development, we isolated 12 distinct thymus-derived thymocyte populations (Fig. 4A) and analyzed them by IR-seq (see Methods). This newly developed method utilizes RNA baits spanning the *Tcra*, *Tcrb*, and *Tcrg* loci to enrich sequencing libraries generated from total cellular DNA extracts comprising both chromosomal DNA and EC DNA (see Methods). This approach suppresses EC size selection bias, allowing quantification of the frequency of J-J, V-V, and V-J excision events.

IR-seq successfully enriches sequences in the target regions (average fold enrichments: *Tcra*, 55 ± 16; *Tcrb*, 47 ± 14; and *Tcrg*, 128 ± 32) (Supplemental Table 3). RF and DP read-pairs were abundant in the T cell developmental libraries and displayed specific enrichment across the *Tcrb*, *Tcrg*, and *Tcra* loci at the expected developmental stages (Supplemental Table 4). In contrast to EC-seq, where small putative Jα-Jα ECs were artificially enriched, normalized coverage in all IR-seq libraries showed no enrichment across the Jα region, confirming that this technique avoids size selection bias (Fig. 4B). Heatmap analysis of RF read-pairs confirmed the presence of Jα-Jα recombinations at developmental stages during which the TCRα chain is rearranged (Fig. 4C). Abundant Jα-Jα RF junctions were detected concurrently with Vα-Jα RFs at an approximate ratio of 1:10 (Fig. 4D). Vα-Vα RFs were also generated, albeit at the lower ratio to Vα-Jα RFs of ∼1:100 (Fig. 4D).

Vα-Jα signal and coding junctions were first observed at the intermediate CD4^+^CD8^+^ thymocyte stage (Supplemental Table 4), when the TCRα chain is generated and a complete αβTCR can be assembled (Fig. 4A; Petrie et al. 1995). The ratio of Vα-Jα signal (RF) and coding (DP) junctions was, as expected, initially equal at the time of recombination (Supplemental Table 4). Upon further maturation, a percentage of thymocytes undergo cell proliferation. As EC DNA is not replicated, the Vα-Jα signal-to-coding junction ratio was progressively diminished, albeit only slightly, as little cellular proliferation occurs following successful TCRα chain rearrangement (Fig. 4E; Supplemental Table 4). Of interest, Jα-Jα ECs showed a marked paucity of corresponding Jα-Jα genomic deletion junctions (mean Jα-Jα RF-to-DP ratio ∼500:1) (Fig. 4E), suggesting that Jα-Jα hybrid ECs are not produced in an identical fashion to Vα-Jα ECs.

To investigate whether J-J ECs were present at T cell antigen receptor loci other than *Tcra*, we next analyzed our IR-seq data sets for the occurrence of Jβ-Jβ or Jγ-Jγ RFs. The first Jβ-Jβ and Jγ-Jγ RF signal junctions were detected at the DN3 stage of mouse T cell development (Fig. 4A; Supplemental Table 4), which is consistent with the expression of the pre-TCR complex at this early developmental check-point (Dudley et al. 1994). Analysis of our EC-seq libraries also revealed enrichment of J-J recombinations at the *Tcrb* and *Igh* loci of our thymic libraries (Supplemental Tables 5, 6), confirming that excision and circularization of J-J genomic regions occur in thymocytes for both T and B cell antigen receptor chain loci. Jα-Jα RFs were also present in human EC-seq libraries prepared from peripheral naïve CD4^+^ and CD8^+^ T cells (Supplemental Table 7; Supplemental Fig. 2), confirming that Jα-Jα hybrid junction formation is not specific to mouse lymphocytes.

### The nature of Jα-Jα 12/12 hybrid junctions

**Figure 5.**
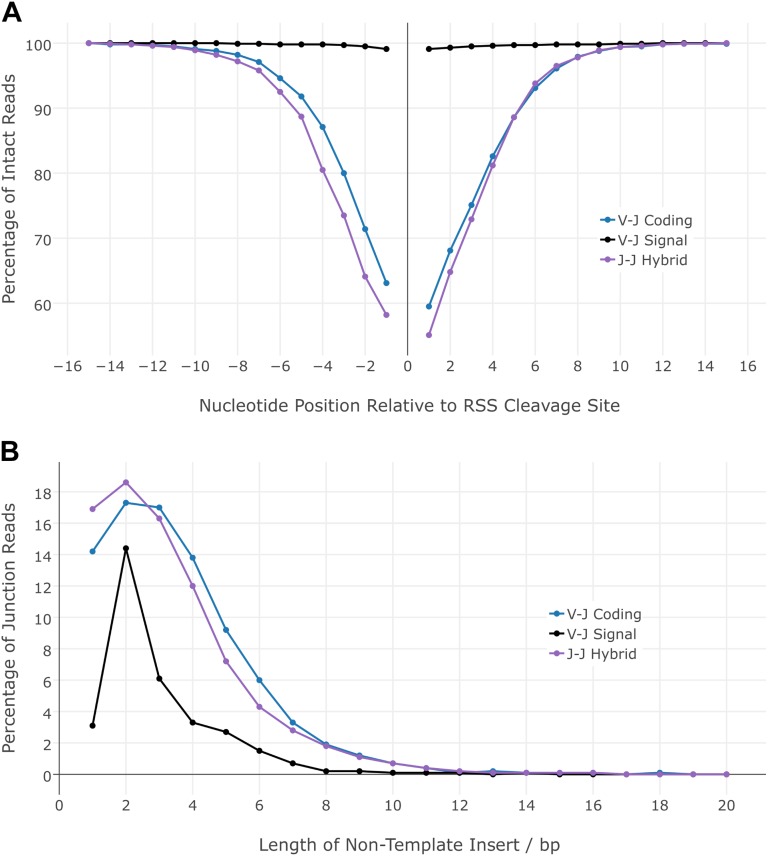
Analysis of Vα-Jα coding, Vα-Jα signal, and Jα-Jα hybrid junctions shows that Jα-Jα hybrids have processing characteristics similar to Vα-Jα coding junctions. (*A*) Frequency of nucleotide deletions flanking the RSS site of cleavage in coding Vα-Jα junctions (blue line), signal Vα-Jα junctions (black line), and hybrid Jα-Jα junctions (purple line). (*B*) Length of nontemplated bases in coding Vα-Jα junctions (blue line), signal Vα-Jα junctions (black line), and hybrid Jα-Jα junctions (purple line).

Previous reports of nonphysiological hybrid junctions indicate variations in the degree of junction processing. These suggest that hybrid junction ends may be processed asymmetrically (Lewis et al. 1988) or not at all (Bogue et al. 1997). To investigate further, we analyzed 13,081 EC-seq or IR-seq junction sequences across 262 different Jα-Jα RF segment combinations. For comparison, we also analyzed 6173 Vα-Jα RF and 5443 Vα-Jα DP complete junction sequences spanning 930 Vα-Jα signal-to-signal and 921 Vα-Jα coding-to-coding segment combinations, respectively. Our data confirmed that Vα-Jα signal joints were resolved with a high degree of substrate conservation, whereas Vα-Jα coding joints exhibited marked deletion of perijunctional nucleotides (Fig. 5A). We found that Jα-Jα 12/12 hybrid signal-to-coding junctions were resolved with similar degrees of deletion to the canonical 23/12 Vα-Jα coding-to-coding joints. Despite the asymmetric nature of Jα-Jα hybrid junctions, the frequency distribution of perijunctional deletions was symmetrical.

Terminal deoxynucleotidyl transferase (TdT) is responsible for the addition of nontemplated “N” base additions during the processing of Vα-Jα coding junctions, a process that adds further diversity to the productive antigen receptor (Desiderio et al. 1984). We compared the presence and length distribution of “N” bases in 13,076 reads containing the full sequence of 262 different Jα-Jα hybrid junctions. Results were compared to the analysis of 11,616 reads containing signal or coding junction sequences spanning >900 different Vα-Jα segment combinations (Fig. 5B). We found that Jα-Jα 12/12 hybrid junctions and Vα-Jα coding junctions displayed highly similar frequencies of “N” base inclusions; “N” bases were present in 82.8% and 85.7% of reads, respectively, whereas only 32.6% of the Vα-Jα signal junctions contained “N” base additions (Fig. 5B). Hence, TdT appears to be active in Jα-Jα 12/12 hybrid junction processing.

## Discussion

We have developed two powerful new tools, EC-seq and IR-seq, for second-generation sequencing-based analyses of genomic recombination events in wild-type immune samples. These sensitive techniques are capable of capturing V(D)J activity from whole immune tissues or from as little as a few thousand sorted cells, allowing detailed profiling of rearrangements at defined time points or in cells with specific developmental phenotypes. To our knowledge, this is the first application of unbiased deep sequencing technology to investigate immune cell–associated rearrangements at the genomic level.

Both methods have independently revealed an unexpected abundance of Jα-Jα and, to a lesser extent, Vα-Vα RF read-pairs that represent the junction sites of Jα-Jα 12/12 hybrid or Vα-Vα 23/23 hybrid excision circle recombinations. The relatively low frequency of Vα-Vα events observed in both EC-seq and IR-seq data sets more likely suggests a physiological, though currently not further identified, difference in the formation of these hybrid events than a size specific sampling bias. We conclude that these EC junctions are frequent physiological events that differ from conventional RAG-mediated recombinations in that they both violate the 12/23 rule and result in a hybrid coding-to-signal junction. This is the first report of high-frequency nonclassical recombination events within antigen receptor loci under physiological conditions in mice and humans. These unprecedented events are both RAG dependent and arise at the same T cell developmental stages as conventional Vα-Jα ECs. They are frequent, accounting for up to 10% of all *Tcra* ECs, and diverse, involving the majority of annotated Jα RSSs. Despite the underlying asymmetry of their constituent ends, they are cleaved and processed similarly to classical 12/23 coding junctions.

**Figure 6.**
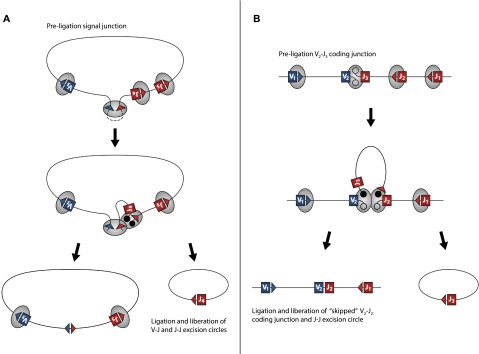
Alternative models of J-J EC production. (*A*) V_2_-J_3_ recombination (see Fig. 1) forms an excision circle pre-ligation intermediate containing variable (blue box) and joining (red box) gene segments with RAG complexes (gray circles) bound at multiple RSS sites (red and blue triangles). Additional synapsis and dsDNA cleavage occurs between an internal J segment RSS and RAG complex bound to either unresolved signal ends or a fully ligated signal junction (as denoted by a dashed line). V-J signal and J-J hybrid junction ends (filled circles) are resolved and liberated as independent ECs. (*B*) V_2_-J_3_ recombination forms a coding junction pre-ligation intermediate with hair-pinned ends (gray circles) containing variable (blue box) and joining (red box) gene segments with RAG complexes (gray circles) bound at multiple RSS sites (red and blue triangles). Additional synapsis and dsDNA cleavage occurs between the RAG complex bound to the unresolved coding end and the flanking J_2_ segment RSS. The initial V_2_-J_3_ coding junction is “skipped,” and a V_2_-J_2_ coding junction is formed along with a J-J hybrid junction containing EC.

Given the abundance of Jα-Jα ECs, we found very few Jα-Jα genomic deletions, suggesting that it is unlikely that they are formed by simple Jα-Jα cleavage, EC circularization, and coding junction re-ligation. Rather, genomic excision in the absence of a detectable deletion junction suggests that the element is either liberated from the terminus of a linear DNA or from a previously resolved coding or signal junction. The latter event is more plausible given that signal junctions retain both 12 bp and 23 bp RSS motifs (Fig. 6). It has been proposed that in addition to the processed V-J pair, RAG recombinase complexes simultaneously engage multiple RSSs flanking the actively processed J element (Schatz and Ji 2011). Hence, RAG complexes internal to an excised fragment may further catalyze the liberation of J-J products from the termini of the EC fragment prior to or even following resolution of a signal junction (Fig. 6A). An alternative model compatible with our data proposes the liberation of J-J ECs from incompletely resolved coding ends (Fig. 6B).

Two conditions need to be met during V(D)J gene recombination in order to optimize the probability of productive recombination: Hybrid junctions must be avoided, and the recombination process must adhere to the 12/23 rule. Our results now show that these conditions are frequently bypassed under physiological conditions. This implies that factors are localized transiently or spatially at restricted subnuclear sites to modulate the stringency of RAG-mediated recombinations. In addition to providing powerful new tools for the analysis of V(D)J mechanisms in immune cells, our report provides data giving new insights into RAG function.

## Methods

### Excision circle (EC)-seq

Thymocytes and splenocytes were freshly isolated from wild-type and *Rag1*-deficient 6- to 8-wk-old C57BL/6 mice (Mombaerts et al. 1992). Cells were dounced in a borosilicate glass mortar (Jencons), centrifuged (800*g* for 5 min at 4°C; Eppendorf 5810R), and resuspended in 1 mL ice-cold nuclear homogenization buffer (Okazaki et al. 1987) by pipetting. After 10 min on ice, nuclei were pelleted by centrifugation (800*g* for 5 min at 4°C), washed in 1 mL nuclear homogenization buffer, and collected by centrifugation (800*g* for 5 min at 4°C). Nuclei were resuspended in 250 μL buffer P1 (Qiagen) and processed using a plasmid purification mini prep kit (Qiagen). Purified excision circles (EC) were eluted in 50 μL 1× Tris-EDTA (TE) and incubated (3 h at 37°C) with 20 units RecBCD (Epicentre) and 20 units T5 (Epicentre) exonucleases in 1× exonuclease buffer supplemented with 1 mM ATP (Epicentre) followed by termination for 20 min at 80°C. EC-DNA was ethanol precipitated with a carrier (Dr. GenTLE, Takara), resuspended in 50 μL 1× TE buffer, and quantified using PicoGreen (Invitrogen) (Parkinson et al. 2011). Illumina-compatible libraries were produced (Parkinson et al. 2011) and sequenced as 100-bp paired-end reads on the Illumina HiSeq 2000 platform.

### Immune region (IR)-seq

Single-cell suspensions of thymi from 6- to 8-wk-old female wild-type and *Rag2*-deficient mice (Shinkai et al. 1992) were enriched for CD4^−^CD8^−^ (double-negative [DN]) cells following depletion of CD4^+^ thymocytes (AutoMACS Pro, Miltenyi Biotec). Viable cells were sorted using specific cell surface markers (Supplemental Tables 12 and 13) on a FACSAria II flow cytometer (BD Biosciences). Genomic DNA from ∼10,000 sorted cells or control brain tissue was purified using a DNA micro kit (Qiagen) and quantified using PicoGreen (Invitrogen). Sequencing libraries were prepared (Parkinson et al. 2011), enriched for target regions using a SureSelect bait library (Agilent) with 2× coverage of *Tcra*, *Tcrb*, and *Tcrg* target regions, and sequenced as 100-bp paired-end reads on the Illumina HiSeq 2000 platform.

### EC-DNA PCR validation of J-J junctions

Amplicons spanning specific Jα-Jα junctions were amplified and sequenced using standard protocols (see Supplemental Methods).

### Flow cytometric sorting of human T cells

Peripheral blood mononuclear cells were sorted using standard procedures (see Supplemental Methods). EC-DNA was extracted from 1 million to 10 million cells, prepared into libraries (Parkinson et al. 2011), and sequenced as 100-bp paired-end reads on the Illumina HiSeq 2000 platform.

### Data analysis

Raw FASTQ libraries were filtered, aligned, relative enrichments calculated, and RF or DP read-pairs identified using our standard bioinformatics pipeline. RF and DP read-pairs were further analyzed for association with active RSS junction sites and used to produce heatmap readouts of their combinatorial usage (see Supplemental Methods).

#### Junction read analysis

Demultiplexed, quality-, and duplicate-filtered 92-bp PE FASTQ data sets from all EC-seq and IR-seq libraries were combined and converted into single-end read format. Individual reads were split informatically into “virtual” paired-end (vPE) FASTQ data sets by isolating the 5′ and 3′ 30 bp using our own software. The resultant library was aligned to mouse genome build 70 (NCBI) using Novoalign v.2.07.00 (Novocraft Technologies). Uniquely mapping RF or DP vPE read-pairs spanning individual Vα-Jα or Jα-Jα coding or signal junctions with no alignment mismatches were identified as described previously. For each coding or signal junction, mapped 30-bp DP or RF vPE read-pairs were extended over the junction site by replacing sequence from the parent 92-bp single-end read until homology with the reference was lost. This empirically derived RSS cleavage site was compared to both conserved heptamer/nonamer motifs and published RSS positions (Lefranc 2008) and adjusted manually as necessary. Additional nontemplated bases were defined as having no homology with either junction flank.

## Data access

Sequencing data generated for this study have been submitted to the European Nucleotide Archive (ENA; http://www.ebi.ac.uk/ena/) under accession number ERP006824.

## Supplementary Material

Supplemental Material
