# Supplementary material for: Violation of the 12/23 rule of genomic V(D)J recombination is common in lymphocytes
Source: Genome Res. 2015 Feb;25(2):226–34. doi: 10.1101/gr.179770.114 (PMC4315296; doi:10.1101/gr.179770.114)
Supplement: Supplemental Material [file supp_25_2_226__index.html]

Violation of the 12/23 rule of genomic V(D)J recombination is common in lymphocytes — Violation of the 12/23 rule of genomic V(D)J recombination is common in lymphocytes — Supplemental Material 

# Violation of the 12/23 rule of genomic V(D)J recombination is common in lymphocytes

## Supplemental Material

**Files in this Data Supplement:**

- Supplemental Material.docx
