# Supplementary material for: Violation of the 12/23 rule of genomic V(D)J recombination is common in lymphocytes
Source: Genome Res. 2015 Feb;25(2):226–34. doi: 10.1101/gr.179770.114 (PMC4315296; doi:10.1101/gr.179770.114)
Supplement: Supplemental Material [file supp_gr.179770.114_Supplemental_Material.doc]

**
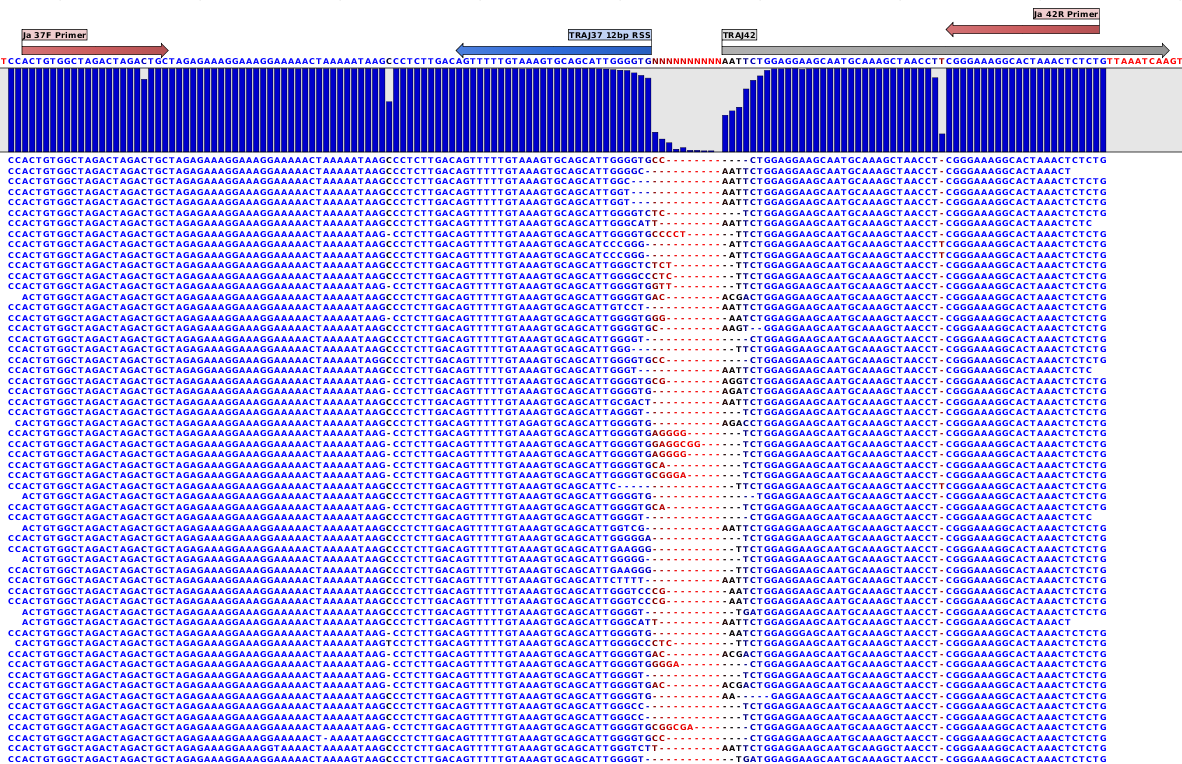
**

A

**
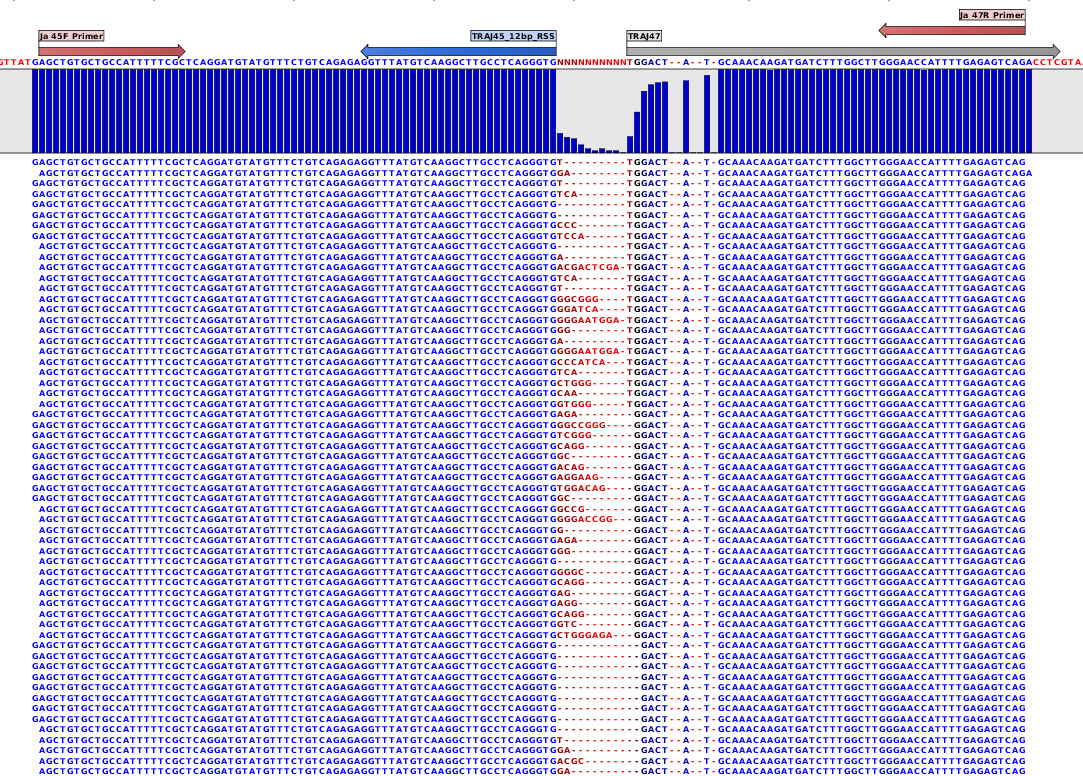
**

B

**
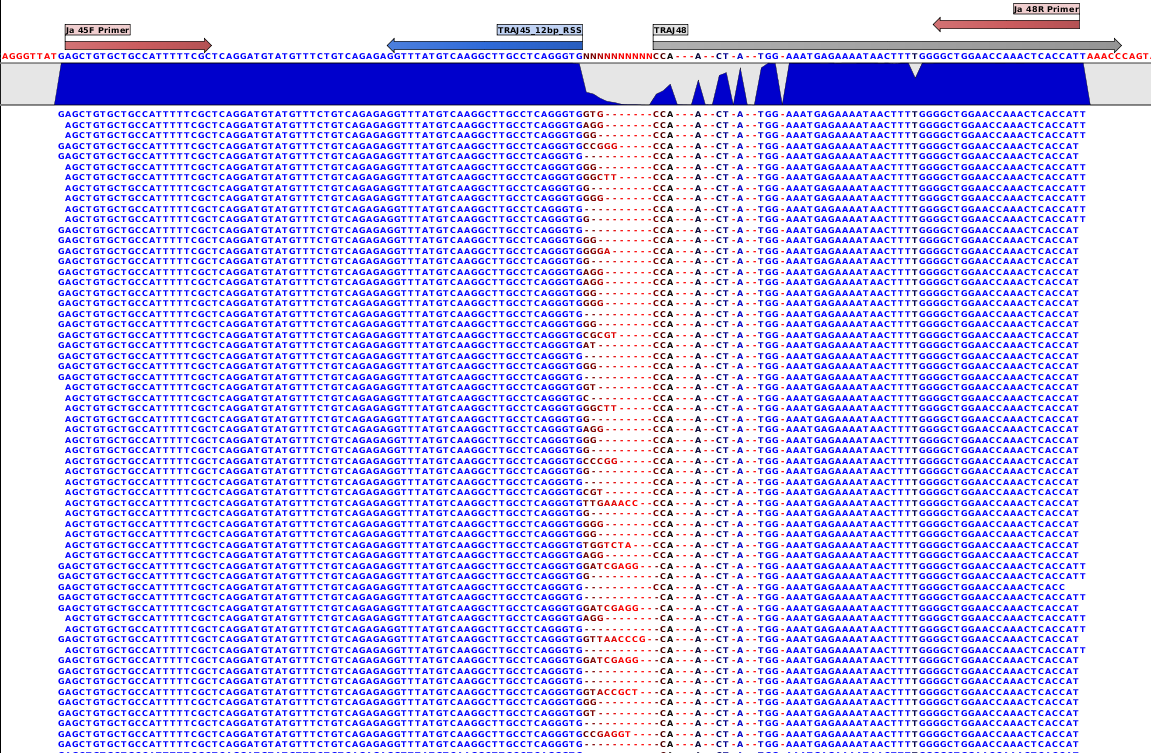
**

C

**
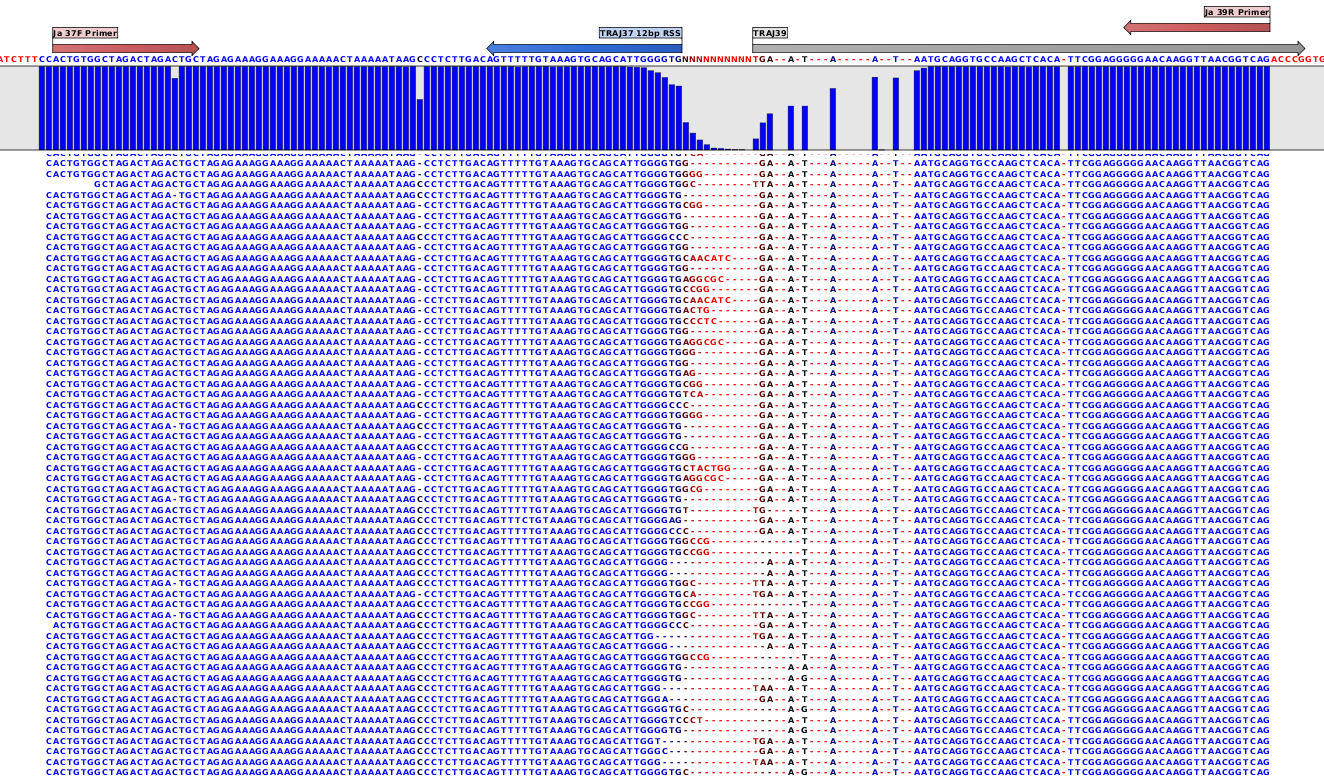
**

D

**Supplementary** **Figure 1.** PCR Validation of selected Ja-Ja junctions in EC DNA shows high diverse sequences at hybrid junction sites. Alignment of sequenced PCR products for (A) *Tcraj37*-*Tcraj42* (B) *Tcraj45*-*Tcraj47* (C) *Tcraj45*-*Tcraj48* (D) *Tcraj37*-*Tcraj39.* Position of PCR primers is shown with red arrows. Blue arrows shows position of 12bp RSS and grey arrow shows position of coding segment. Blue nucleotides denote highly conserved sequences with red and black denoting less conserved nucleotides.

**
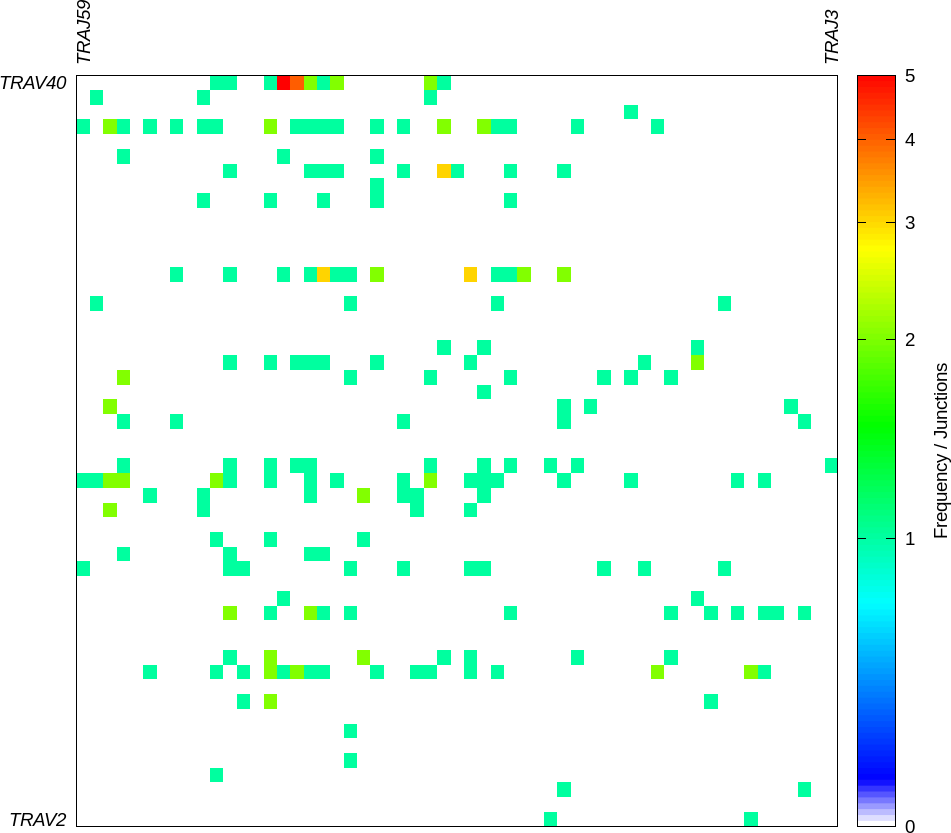

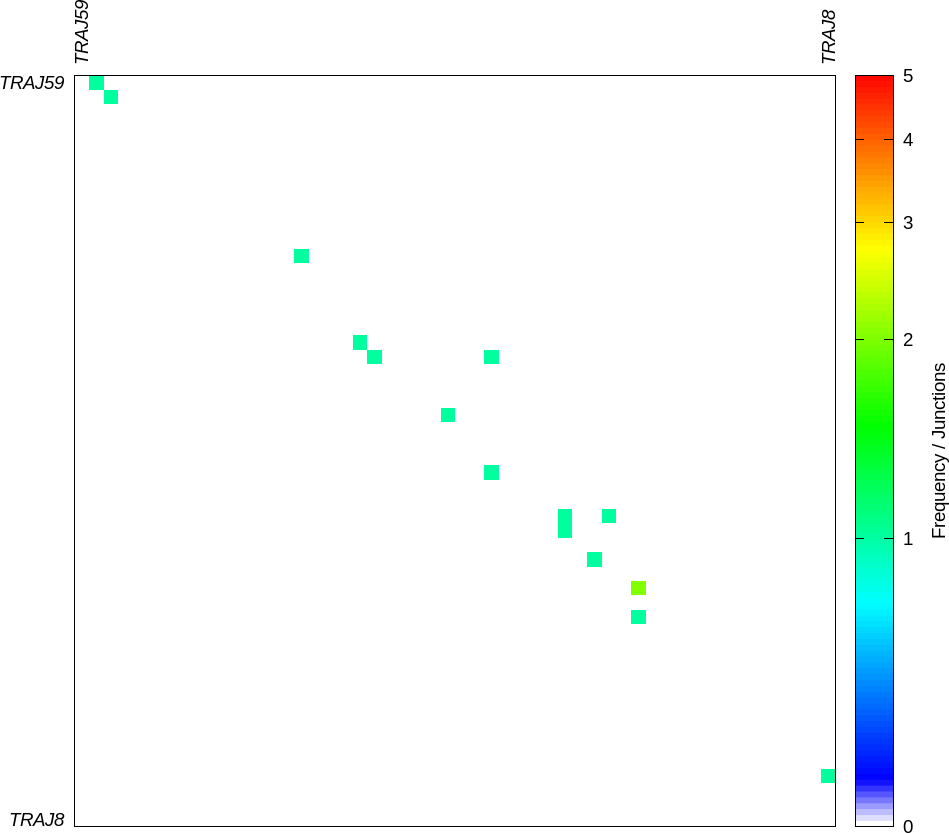
**

A

B

**Supplementary** **Figure 2.** EC-seq captures human Vα-Jα and Jα-Jα ECs from circulating immune cells. (A) Heat-map analysis of 243 Vα-Jα ECs or (B) Sixteen Jα-Jα ECs from human EC-seq libraries.

**Supplemental Table 1.** EC-seq enriches all sequenced material (AA), circle junctions (RF), and deletion junctions (DP) in perfectly aligned read-pairs across the mouse *Tcra* locus in three individual adult whole thymus or spleen preparations. Control region is size, chromosome and mappability matched to the *Tcra* region.


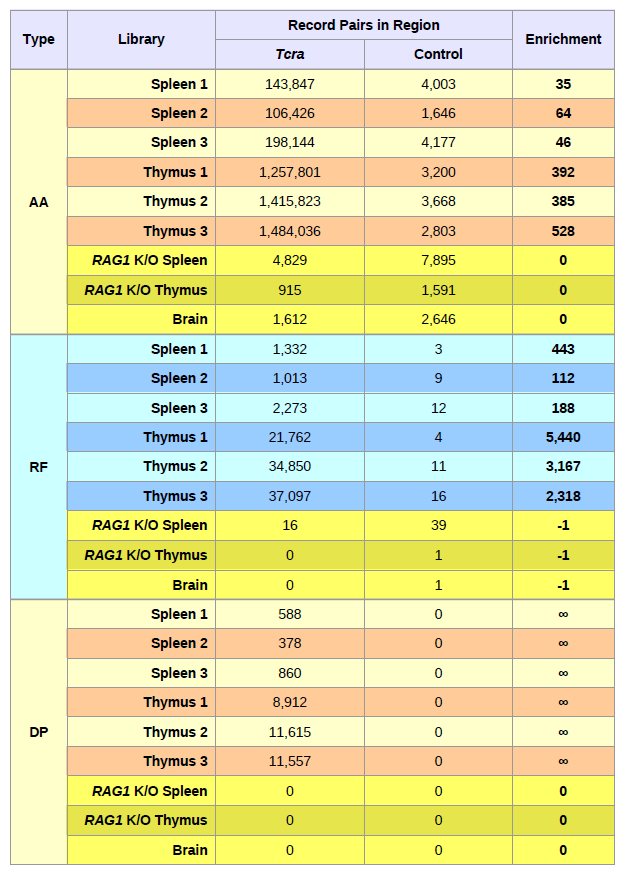


**Supplemental Table 2.** >95% of Vα-Jα coding junction RF reads and >93% of Vα-Jα signal junction DP reads map within 300bp of a known RSS junction across the *Tcra* locus in three replicate thymic and splenic EC-seq libraries.


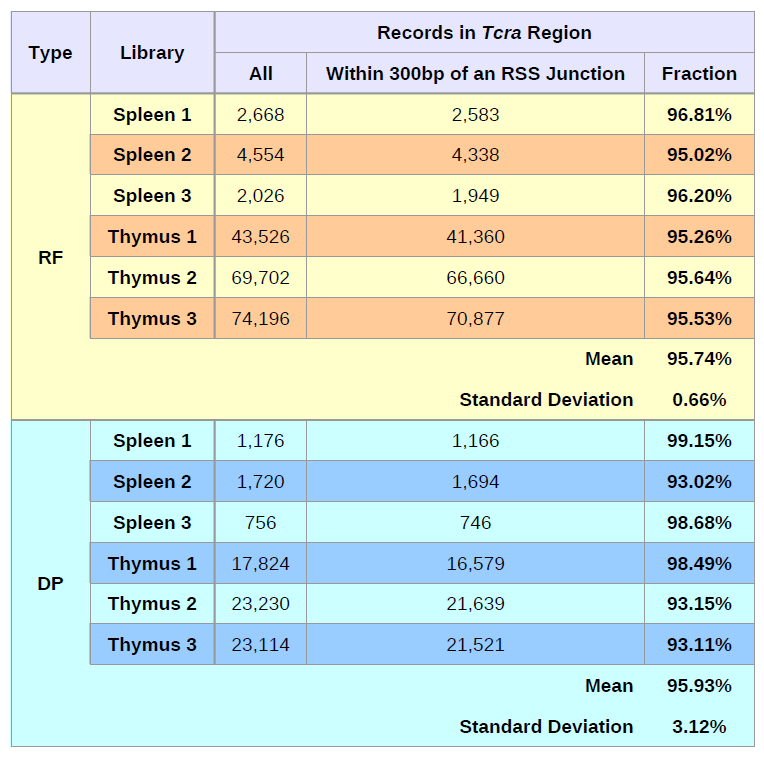


**Supplemental Table 3.** IR-seq enriches material across *Tcra*, *Tcrb* and *Tcrg* compared to size and chromosome matched non-baited control regions.


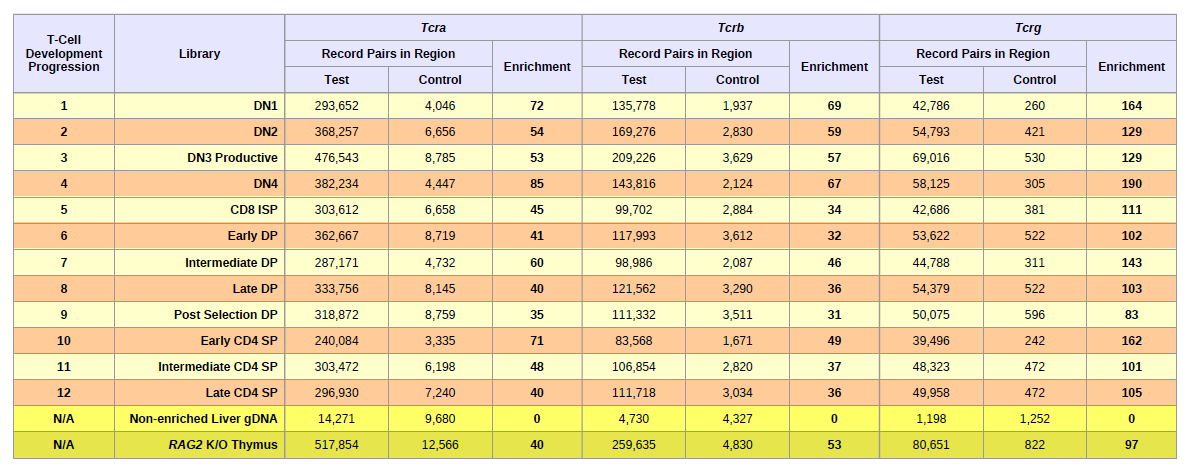


**Supplemental Table 4.** IR-seq enriches RF and DP read-pairs across Tcra, Tcrb and Tcrg compared to RF and DP activity in matched regions of Rag2-deficient whole thymus. Numbers of RF and DP reads are normalised per 10 million input library paired-end reads.


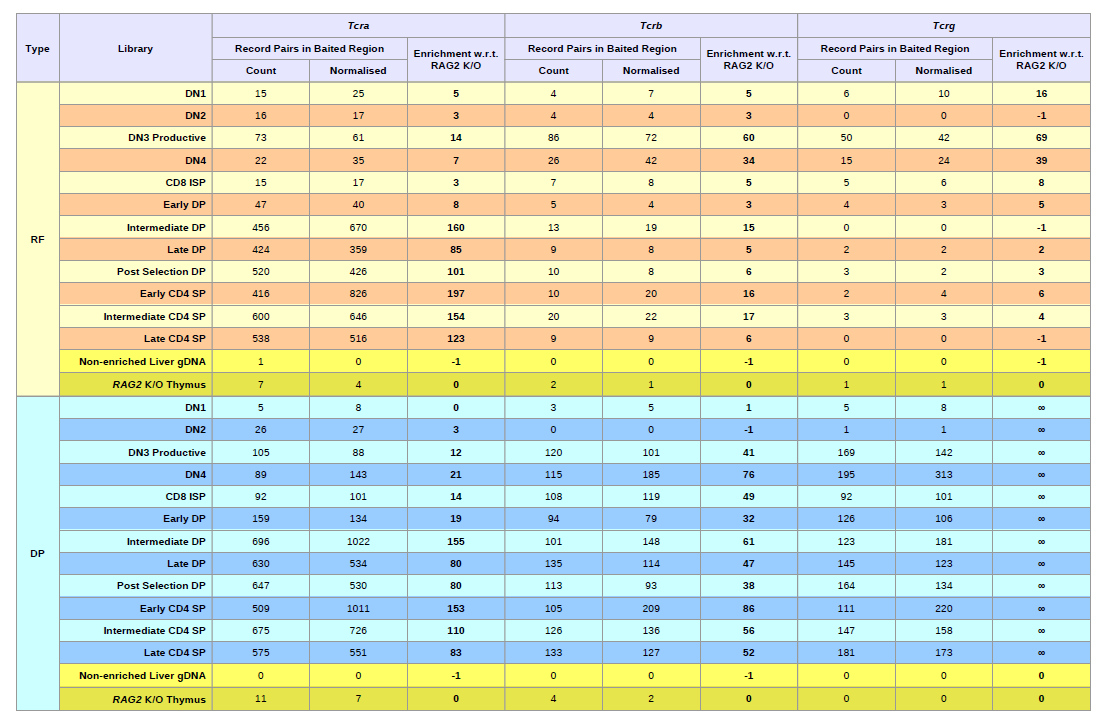


**Supplemental Table 5.** EC-seq enrichments for all material (AA), circle junctions (RFs) and deletion junctions (DPs) across *Tcra*, *Tcrb*, *Tcrg*, *Igh*, *Igl* and *Igk* in three thymic and three splenic libraries.

**
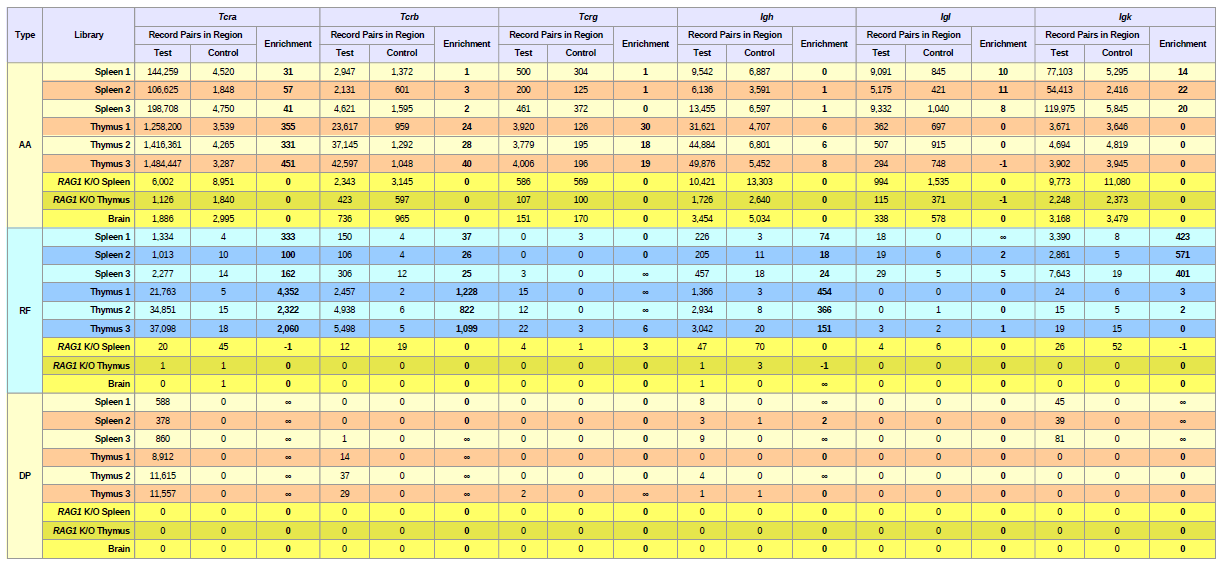
**

**Supplemental Table 6.** Analysis of V-V, V-J and J-J EC sub-types produced across *Tcra*, *Tcrb*, *Tcrg*, *Igh*, *Igl* and *Igk* in three thymic and three splenic libraries shows that J-J ECs are produced by *Tcra*, *Tcrb*, *Igh* and *Igk* in our datasets.

**
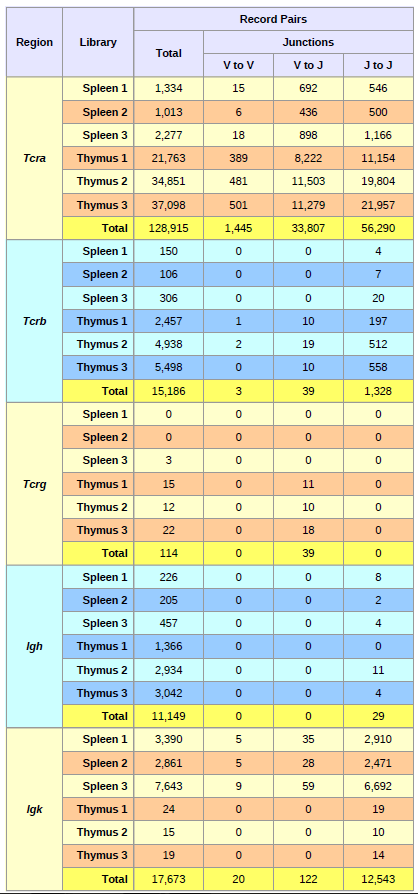
**

**Supplemental Table 7.** EC-seq of human peripheral T cells shows enrichment of immune region-associated sequenced material (AAs), circle junctions (RFs) and deletion junctions (DPs), and reveals the presence of J-J EC sub-types across the *TRA* locus.


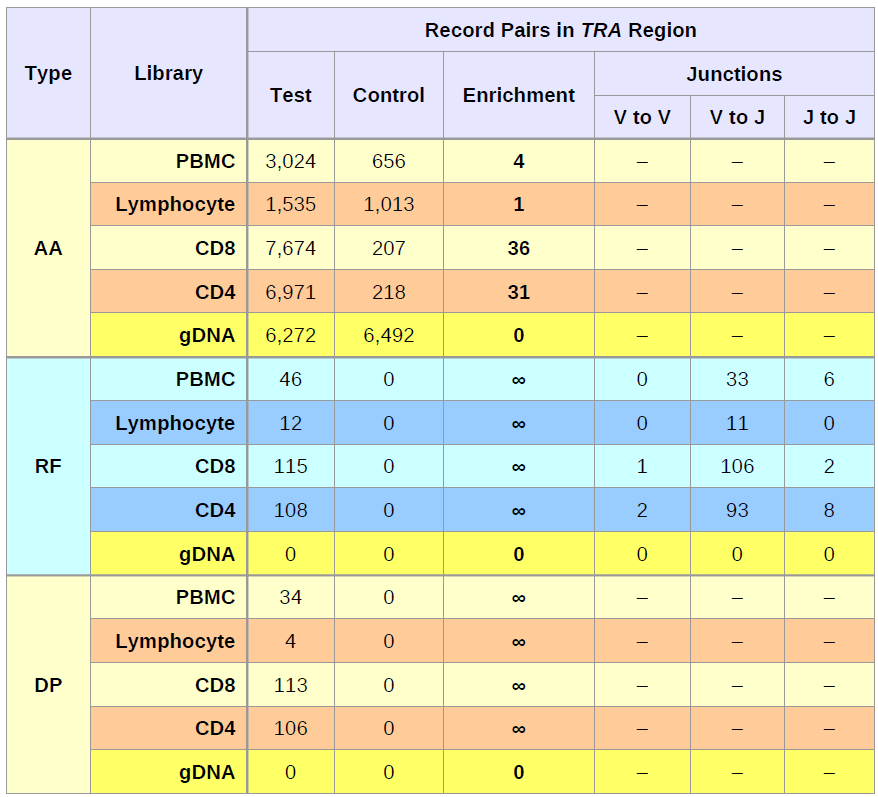


**Supplementary Table 8.** Oligonucleotide sequences used to amplify Jα-Jα junctions.

| Oligo ID | Sequence |
| --- | --- |
| Jα 37f | ACTGTGGCTAGACTAGACTGC |
| Jα 39f | CCTTCCAGAAGATCTGTAGGG |
| Jα 45f | AGCTGTGCTGCCATTTTTCGC |
| Jα 47f | GTCCCAAATGTAAAGCAGGGG |
| Jα 39r | CTGACCGTTAACCTTGTTCCC |
| Jα 40r | AACCTTCAGTCTGGTACCTGC |
| Jα 42r | GAGAGTTTAGTGCCTTTCCCG |
| Jα 47r | CTGACTCTCAAAATGGTTCCC |
| Jα 48r | ATGGTGAGTTTGGTTCCAGCC |

**Supplementary Table 9.** Oligonucleotide combinations used to amplify Jα-Jα EC junctions

| Primer 1 | Primer 2 | Predicted product size |
| --- | --- | --- |
| Jα 37f | Jα 39r | 147 |
| Jα 37f | Jα 40r | 145 |
| Jα 37f | Jα 42r | 143 |
| Jα 39f | Jα 40r | 127 |
| Jα 45f | Jα 47r | 127 |
| Jα 45f | Jα 48r | 126 |
| Jα 47f | Jα 48r | 176 |

**Supplementary Table 10.** Oligonucleotides used in PGM library synthesis

| Oligo ID | Sequence |
| --- | --- |
| PGM P1 PCR adapter oligo 1 | CCACTACGCCTCCGCTTTCCTCTCTATGGGCAGTCGGTGATCCTCAGC*T |
| PGM P1 PCR adapter oligo 2 | /5phos/GCTGAGGATCACCGACTGCCCATAGAGAGGAAAGCGGAGGCGTAGTGG*T*T |
| PGM A PCR adapter oligo 1 | /5phos/CTGAGTCGGAGACACGCAGGGATGAGATGG*T*T |
| PGM A PCR adapter oligo 2 | CCATCTCATCCCTGCGTGTCTCCGACTCAG*T |
| PGM P1 primer | CCACTACGCCTCCGCTTTCCT |
| PGM A primer | CCATCTCATCCCTGCGTGTC |

**Supplementary Table 11.** Human and mouse immune region locations

|  |  | Test region | | | Control region | | |
| --- | --- | --- | --- | --- | --- | --- | --- |
| Locus | Chr | Start | End | Size | Start | End | Size |
| *Tcra* | mm14 | 52,181,000 | 54,230,500 | 2,049,500 | 61,000,000 | 63,049,500 | 2,049,500 |
| *Tcrb* | mm06 | 40,891,088 | 41,560,900 | 669,812 | 30,891,088 | 31,560,900 | 669,812 |
| *Tcrg* | mm13 | 19,177,500 | 19,353,300 | 175,800 | 17,177,500 | 17,353,300 | 175,800 |
| *Igh* | mm12 | 113,208,768 | 116,050,461 | 2,841,693 | 103,208,768 | 106,050,461 | 2,841,693 |
| *Igl* | mm16 | 18,985,017 | 19,310,844 | 325,827 | 17,985,017 | 18,310,844 | 325,827 |
| *Igk* | mm06 | 67,555,630 | 70,776,757 | 3,221,127 | 57,555,630 | 60,776,757 | 3,221,127 |
| *TRA* | hs14 | 22,087,049 | 23,023,543 | 936,494 | 32,087,049 | 33,023,543 | 936,494 |
| *TRB* | hs07 | 141,992,985 | 142,515,565 | 522,580 | 131,992,985 | 132,515,565 | 522,580 |
| *TRG* | hs07 | 38,279,075 | 38,408,124 | 129,049 | 28,279,075 | 28,408,124 | 129,049 |
| *IGH* | hs14 | 106,048,945 | 107,294,480 | 1,245,535 | 96,048,945 | 97,294,480 | 1,245,535 |
| *IGL* | hs22 | 22,377,392 | 23,271,112 | 893,720 | 32,377,392 | 33,271,112 | 893,720 |
| *IGK* | hs02 | 89,142,669 | 90,308,304 | 1,165,635 | 79,142,669 | 80,308,304 | 1,165,635 |

**Supplementary Table 12.** Definition of mouse T-cell ontogeny by antibody reactivity

| **Ontogenic stage** | **ID** | **Surface expression** |
| --- | --- | --- |
| Double Negative 1 | DN1 | Lin- CD44+ CD25- c-kit+ |
| Double Negative 2 | DN2 | Lin- CD44+ CD25+ c-kit+ |
| Double Negative 3  (non-productive TCRβ) | DN3np | Lin- CD44low CD25+ c-kitlow CD71- |
| Double Negative 3 (productive) | DN3 | Lin- CD44low CD25+ c-kitlow CD71+ |
| Double Negative 4 | DN4 | Lin- CD44low CD25- c-kitlow CD71+ |
| CD8 ISP | CD8 ISP | TCRαβ- CD4- CD8+ CD71+ CD69- |
| Early (pre-selection)  Double positive | eDP | TCRαβ- CD4+ CD8+ CD71+ CD69- |
| Intermediate Double Positive | intDP | TCRαβ- CD4+ CD8+ CD71- CD69- |
| Late Double Positive | IDP | TCRαβint CD4+ CD8+ CD71- CD69- |
| Post-selection  Double Positive | psDP | TCRαβint CD4+ CD8+ CD71- CD69+ CD25- |
| Early CD4 Single Positive | eCD4 | TCRαβint CD4+ CD8- CD71- CD69+ CD25- |
| Intermediate CD4 Single Positive | IntCD4 | TCRαβhigh CD4+ CD8- CD71- CD69+ CD25- |
| Late Single Positive | ICD4 | TCRαβhigh CD4+ CD8- CD71- CD69- CD25- |

**Supplementary Table 13.** Mouse antibodies used

| Antibody | Conjugate | Source | Clone |
| --- | --- | --- | --- |
| CD44 | PE | Biolegend | IM7 |
| c-kit | APC | Biolegend | 2B8 |
| CD25 | FITC | Biolegend | PC61 |
| CD71 | PE-Cy7 | Biolegend | RI7217 |
| TCRαβ | Biotinylated | Biolegend | H57-597 |
| CD4 | APC-Cy7 | Biolegend | GK1.5 |
| CD8 | A700 | Biolegend | 53-6.7 |
| CD25 | PerCP-Cy5.5 | Biolegend | PC61 |
| CD69 | FITC | Biolegend | H1.2F3 |
| TCRαβ | PE | Biolegend | H57-597 |
| CD4 | Biotinylated | Biolegend | GK1.5 |
| Streptavadin | APC-Cy7 | Biolegend |  |
| TCRgd | Biotinylated | Biolegend | GL3 |
| CD8 | Biotinylated | Biolegend | 53-6.7 |
| CD3 | Biotinylated | Biolegend | 145-2C11 |
| Gr1 | Biotinylated | Biolegend | RB6-8C5 |
| F4/80 | Biotinylated | Biolegend | BM8 |
| CD19 | Biotinylated | Biolegend | 6D5 |
| CD11b | Biotinylated | Biolegend | M1/70 |
| CD49b | Biotinylated | Biolegend | DX5 |
| TER-119 | Biotinylated | Biolegend | TER-119 |

**Supplementary Table 14.** Human antibodies used

| Antibody | Conjugate | Source | Clone |
| --- | --- | --- | --- |
| CD14 | Pacific Blue | Caltag/Life Technologies | TüK4 |
| CD19 | Pacific Blue | Caltag/Life Technologies | SJ25-C1 |
| CD3 | APC-H7 | BD | SK7 |
| CD4 | PE-Cy5.5 | Caltag/Life Technologies | S3.5 |
| CD8 | PE-Cy7 | BD Pharmingen | RPA-T8 |
| CD45RA | ECD | Beckman Coulter | 2H4LDH11LDB9 |
| CD27 | PE-Cy7 | Beckman Coulter | 1A4CD27 |

**Supplementary Table 15.** Vα and Jα elements excluded from Vα-Jα EC analysis due to ‘inactivity’

| Excluded Vα elements | | | Excluded Jα elements | |
| --- | --- | --- | --- | --- |
| *Tcrav1*  *Tcrav3-1*  *Tcrav4-1*  *Tcrav10D*  *Tcrav6N-5*  *Tcrav11N*  *Tcrav6-5* | *Tcrav5-2*  *Tcrav12-4*  *Tcrav17*  *Tcrav7D-2*  *Tcrav4D-2*  *Tcrav3-2*  *Tcrav15-1/dv6-1* | *Tcrav6D-5*  *Tcrav15N-3*  *Tcrav7-2*  *Tcrav4-2*  *Tcrav18*  *Tcrav22*  *Tcrav23* | *Tcraj51*  *Tcraj29*  *Tcraj25*  *Tcraj3*  *Tcraj1*  *Tcraj59* | *Tcraj55*  *Tcraj54*  *Tcraj20*  *Tcraj14*  *Tcraj8* |

**Supplementary Table 16.** Vα and Jα elements excluded from Vα-Jα EC analysis by mappability

| Excluded Vα elements  with mappability <0.33 | | | Excluded Jα elements with mappability <0.5 |
| --- | --- | --- | --- |
| *Tcrav6D-6*  *Tcrav7D-5*  *Tcrav12D-1*  *Tcrav14D-1*  *Tcrav15D-1/DV6D-1*  *Tcrav3D-1*  *Tcrav9D-2*  *Tcrav4D-3*  *Tcrav5D-2*  *Tcrav12D-2*  *Tcrav9D-3*  *Tcrav5D-3*  *Tcrav12D-3*  *Tcrav13D-2*  *Tcrav14D-2*  *Tcrav15D-2/DV6-2*  *Tcrav3D-3*  *Tcrav9D-4*  *Tcrav4D-4*  *Tcrav6D-7*  *Tcrav7D-6* | *Tcrav7N-5*  *Tcrav12N-1*  *Tcrav14N-1*  *Tcrav15N-1*  *Tcrav3N-2*  *Tcrav9N-2*  *Tcrav4N-3*  *TcravV12N-2*  *Tcrav9N-3*  *Tcrav5N-3*  *Tcrav12N-3*  *Tcrav13N-2*  *Tcrav14N-2*  *Tcrav15N-2*  *Tcrav3N-3*  *Tcrav4N-4*  *Tcrav6N-7*  *Tcrav7N-6*  *Tcrav6-6*  *Tcrav5D-4*  *Tcrav5N-4*  *Tcrav13D-1*  *Tcrav13N-1* | *Tcrav13N-3*  *Tcrav9N-4*  *Tcrav9D-1*  *Tcrav16D/DV11*  *Tcrav9N-1*  *Tcrav11*  *Tcrav13-3*  *Tcrav13D-3*  *Tcrav11D*  *Tcrav8-1*  *Tcrav9-1*  *Tcrav8D-1*  *Tcrav16N*  *Tcrav15-3*  *Tcrav13D-4*  *Tcrav3-3*  *Tcrav9-4*  *Tcrav14N-3*  *Tcrav13-4/DV7*  *Tcrav15-2/DV6-2*  *Tcrav15D-3*  *Tcrav8D-2* |  |

**Supplemental Methods**

**EC-DNA PCR validation of J-J junctions**

Oligonucleotides were designed to produce 100-200 bp amplicons spanning Jα-Jα junctions (Supplementary Tables 8 and 9). Amplification reactions (20 μL) contained 1x OneTaq quick load (New England Biolabs), 10 pmol forward primer, 10 pmol reverse primer and 250 pg EC-DNA, denatured at 98^o^C for 10 min, cycled 30x using 98^o^C for 30s, 60^o^C for 30s and 72^o^C for 15s, then extended at 72^o^C for 5 min. Products were visualised on 3% NuSieve Agarose (GTG), excised and recovered using a gel extraction kit (Zymo). Products were pooled and used to make sequencing libraries and run on a Personal Genome Machine 314 chip (Ion Torrent). Data was imported into CLC Genomics Workbench v6.0.2 (CLC Bio) and aligned to Mouse Genome Assembly Build 38 release 70 (NCBI).

**Flow cytometric sorting of human T cells**

Peripheral blood mononuclear cells (PBMCs) were isolated from fresh human blood by density gradient centrifugation (Lymphoprep, Axis-Shield), washed twice with PBS and incubated with LIVE/DEAD Fixable Violet (Life Technologies) for 10 min at room temperature, after which they were stained with antibodies (Supplementary Table 14) and stored at 4^o^C overnight in RPMI medium with 10% fetal bovine serum, penicillin/streptomycin, glutamine and HEPES (Sigma). Cells were then sorted using a modified FACSAria II flow cytometer (BD Biosciences). EC-DNA was extracted from 1-10 million cells, prepared into libraries (Parkinson et al. 2011) and sequenced as 100 bp paired-end reads on the Illumina HiSeq 2000 platform.

**Data analysis**

Raw FASTQ libraries were filtered to remove reads containing Phred <20 bases, demultiplexed, trimmed to 43 bp and duplicate-filtered using our own software. Library quality was assessed using the FastQC v0.10.1 software package (Andrews 2010). Filtered datasets were aligned to either Mouse Genome Build 38 release 70 (NCBI) or Human Build 37 release 61 (NCBI) using Novoalign v.2.07.00 software (Novocraft Technologies). Uniquely mapping reads with no alignment mismatches were isolated and further divided into RF or DP sub-libraries using our own software. RFs were defined as read-pairs aligning without mismatch to the reference genome in a ‘reverse’ before ‘forward’ read configuration and separated by implied mapping distances of 100 bp to 3 Mbp. DPs were defined as read-pairs aligning without mismatch to the reference genome in a ‘forward’ before ‘reverse’ read configuration and separated by implied mapping distances of 1 kbp to 3 Mbp. SAM records or FASTQ files were visualised in CLC Genomics Workbench v6.0.2 (CLC Bio) or gnuplot v4.4.

EC-seq relative fold enrichment for specific genomic regions (Supplementary Table 11) were calculated using our own software as follows:

(read count in test region – read count in control region)

read count in control region

where control regions are size and chromosome matched regions of equivalent mappability as judged by a test region total read count/control region total read count ratio between 0.8 and 1.2 in a non-enriched gDNA library.

*RF associations with RSS junction sites*

Genomic mapping positions of *Tcra* locus RF read-pairs were adjusted to relative values to their nearest RSS using our own software. Relative RF datasets were overlaid and read associations to RSS junctions calculated and visualised (gnuplot v4.4) as percentage reads within 300 bp windows flanking RSS sites.

*Excluding inactive RSS junctions*

EC-seq RF data was pooled from all mouse EC-seq datasets. Vα or Jα elements were deemed inactive and excluded from further analysis if fewer than seven reads mapped within 300 bp flanks of their RSS’s (Supplementary Table 15).

*Tcra RSS junction mappability*

Mappability scores were computed for 300 bp windows flanking Vα and Jα elements for the Mouse or Human genome as defined with Genbank annotations and published RSS positions (Lefranc 2013). All potential 43 bp positions from each window were assessed for unique mappability by aligning to the complete Mouse or Human genomes under high stringency conditions. Mappability scores were generated as a ratio of uniquely mapping to non-uniquely mapping constituent 43 bp sub-reads for each 300 bp window. For Vα-Jα EC analysis, elements were excluded if the mappability ratio of 300 bp regions distal to the Vα RSS site or proximal to the Jα RSS site fell below 0.33 or 0.5 respectively (Supplementary Table 16). For Jα-Jα EC analysis, elements were excluded if the mappability ratio of 300 bp regions either distal or proximal to the Jα RSS site fell below 0.5. Based on this filter *Tcraj26, Tcraj19, Tcraj36, Tcraj24, Tcraj60 and Tcraj46* were excluded from these analyses.

*Heat-map calculations*

Mappability and activity filtered RF read-pairs were interrogated further using a combinatorial matrix of RSS coordinates to produce a frequency usage map of pair-wise permutations of V and J elements using our own software and visualised graphical with gnuplot v4.4.

*IR-seq coverage*

Mapped read-pairs were counted for defined *Tcra* inter-RSS bins to provide coverage maps across the entire locus. Coverage was normalised using the pre-recombination stage DN1 library dataset.
